# Drug-eluting beads transarterial chemoembolization with CalliSpheres microspheres for treatment of unresectable intrahepatic cholangiocarcinoma

**DOI:** 10.7150/jca.39410

**Published:** 2020-05-18

**Authors:** Tan-Yang Zhou, Guan-Hui Zhou, Yue-Lin Zhang, Chun-Hui Nie, Tong-Yin Zhu, Hong-Liang Wang, Sheng-Qun Chen, Bao-Quan Wang, Zi-Niu Yu, Li-Ming Wu, Shu-Sen Zheng, Jun-Hui Sun

**Affiliations:** 1 Hepatobiliary and Pancreatic Interventional Treatment Center, Division of Hepatobiliary and Pancreatic Surgery, The First Affiliated Hospital, College of Medicine, Zhejiang University, Hangzhou 310003, Zhejiang Province, China; 2 Zhejiang Provincial Research Center for Diagnosis and Treatment of Hepatobiliary Diseases, Hangzhou 310003, Zhejiang Province, China; 3 Zhejiang Clinical Research Center of Hepatobiliary and Pancreatic Diseases, Hangzhou 310003, Zhejiang Province, China

**Keywords:** Transarterial chemoembolization, Intrahepatic cholangiocarcinoma, Drug-eluting beads, Prognostic factors, CalliSpheres microspheres

## Abstract

**Objective**: This study aimed to evaluate the efficacy and safety of doxorubicin-loaded drug-eluting beads transarterial chemoembolization (DEB-TACE) with CalliSpheres microspheres (CSM) in treating unresectable intrahepatic cholangiocarcinoma (ICC).

**Methods**: 88 unresectable ICC patients who received DEB-TACE treatment with CSM were retrospectively enrolled in this study. Information about treatment response, survival and adverse events were collected. The Kaplan-Meier curve was used to evaluate progression-free survival (PFS) and overall survival (OS), and factors affecting OS were determined by Cox's proportional hazards regression model.

**Results**: Tumor response of the whole sample of 88 patients was partial response (PR) in 58 (65.9%) patients, stable disease (SD) in 19 (21.6%) and progressive disease (PD) in 11 (12.5%) at one month after therapy, with no complete responses (CR). The median PFS and OS were 3.0 months and 9.0 months respectively. Cox's proportional hazards regression analysis disclosed that subsequent treatment was an independent favorable prognostic factor, while cholangiectasis, extensive intrahepatic tumor burden and extrahepatic metastasis were the three prognostic factors associated with poor survival in ICC patients. Besides, common adverse events included nausea/vomiting, abdominal pain and transient elevation of liver transaminase in patients treated by DEB-TACE with CSM.

**Conclusion**: DEB-TACE with CSM is safe and well-tolerated for unresectable ICC patients, with a low complication rate and a relative benefit in terms of survival. Subsequent treatments including systemic/loco-regional treatments is an independent favorable prognostic factor, but cholangiectasis, extensive intrahepatic tumor burden and extrahepatic metastases are the three prognostic factors associated with poor survival.

## Introduction

Intrahepatic cholangiocarcinoma (ICC) is a fatal primary liver cancer arising from the epithelial lining of the peripheral intrahepatic bile duct epithelium 1. As the second most common type of primary liver cancer, ICC has more aggressive tumor behaviors than hepatocellular carcinoma (HCC), resulting in a devastating prognosis and high mortality rate^2^. Liver resection is the mainstay treatment of this disease 1. Unfortunately, most patients with ICC are found to have locally advanced or metastatic disease at the time of diagnosis. Even after resection with curative intent, early recurrence and metastasis are very common and the prognosis remains very poor 3-4. Therefore, it is of great necessity to investigate in non-surgical and locoregional treatments to provide survival benefits for unresectable ICC patients.

Transarterial chemoembolization (TACE), as one of the most commonly applied non-surgical locoregional treatments for liver cancers, has been illustrated by accumulating clinical researches to effectively diminish tumor tissue and improve the prognosis of liver cancer patients 5-7. Traditionally, the drug carrier used in TACE is lipiodol, which has strong liquidity and relatively weak sustained-release effect resulting in systemic toxicity. Therefore, other interventional embolic materials such as drug-eluting beads (DEB) have been investigated as replacements for lipiodol to better load and release drugs in TACE treatment 8.

As a novel type of drug-eluting microspheres, CalliSpheres microspheres (CSM) is the first microsphere product independently researched and developed in China, and it has been applied in the treatment of Chinese HCC patients with quite promising clinical outcomes 8. However, CSM has just been launched to the market and the number of studies about DEB-TACE with CSM in liver cancers is still small. Besides, there is currently no research on the treatment efficacy of DEB-TACE with CSM in unresectable ICC patients as far as we know. Therefore, we summarized the short-term efficacy and perioperative complications of this therapeutic scheme by collecting and analyzing the clinical data of 88 consecutive patients with unresectable ICC who accepted DEB-TACE treatment with CSM in our center from November 2015 and May 2018, with an attempt to further clarify its safety and effectiveness as well as factors influencing the prognosis.

## Methods

### Patients

From November 2015 and May 2018, 88 patients with unresectable ICC who received DEB-TACE in the First Affiliated Hospital, College of Medicine, Zhejiang University were included in this retrospectively study. The inclusion criteria were: (1) diagnosed as ICC confirmed by liver biopsy (58 cases) or postoperative pathological examinations (30 cases); (2) underwent DEB-TACE treatment using CSM; (3) clinical data and follow-up records were completely reserved and accessible. Patients who complicated with other malignancies or whose medical records were incomplete were excluded from the study. The present study was approved by the Institutional Review Board of the First Affiliated Hospital, College of Medicine, Zhejiang University, and written informed consents were obtained from all the patients or their statutory guardians.

### Data collection

Patients' baseline demographic and clinical characteristics were collected from electronic medical records, including age, gender, ECOG performance status, Child-Pugh classification, laboratory and pathologic parameters, radiological characteristics and treatment courses.

### Process of drug loading

Before the initiation of DEB-TACE, the concentrated solution of epirubicin was prepared with 4 ml of sterile water for injection and 80 mg of epirubicin hydrochloride, and the concentration was 20 mg/ml. Then the CSM (diameter 100-300μm) were loaded with epirubicin as follows: firstly, the CSM and sterile water were extracted by a 20 mL syringe and inverted placed for 5 min until the CSM were totally precipitated, then pushed out the supernatant liquor. Subsequently, the concentrated solution of epirubicin was mixed with the CSM using a tee joint and then stored by a syringe, and the syringe containing the mixture of CSM and epirubicin solution was placed at room temperature and shaken gently every 5 min until almost all epirubicin were loaded (loading time more than 30 min). After that, the nonionic contrast medium (iodixanol [320 mg I/mL], Jiangsu Hengrui Medicine, Jiangsu, China) was added into the mixture as a 1:1 ratio and the mixture were kept still for 5 min for further application.

### Procedures of DEB-TACE

Before DEB-TACE, the examinations of whole blood, routine biochemistry, coagulation function and serum tumor markers were performed, and contrast-enhanced abdominal computerized tomography (CT) or gadoxetic acid-enhanced liver magnetic resonance imaging (MRI) was performed for all patients. After local anesthesia, angiography was performed to detect tumor feeders using Seldinger's technique through a transfemoral approach, which included the hepatic artery, superior mesenteric artery and the inferior phrenic artery, as well as other visceral arteries if necessary. When the tumor feeders were identified, 2.4-F to 2.8-F microcatheter was recommended to use for a segment or subsegment super-selective catheterization of tumor feeders. Subsequently, the mixture of CSM and nonionic contrast medium was injected into the tumor feeders through the microcatheter at a speed of 1 mL/min. When blood flow slowed or the small branch of portal vein appeared, the embolization stopped. When the angiography showed tumors staining disappeared or disappeared mostly, the operation finished. Embospheres or PVA particles could be added if necessary. All patients were treated with symptomatic support such as routine liver protection, pain relief, antiemetics and prophylactic anti-infection after DEB-TACE.

### Assessment and follow up

Contrast-enhanced abdominal CT or gadoxetic acid-enhanced liver MRI examination was performed one month after the first DEB-TACE treatment and then every 4-6weeks to assess response to treatment. The treatment response including complete response (CR), partial response (PR), stable disease (SD) and progressive disease (PD) was assessed according to the modified Response Evaluation Criteria in Solid Tumors (mRECIST)^9^. All adverse events(AEs) were recorded and evaluated using the five grade Common Therapy Evaluation Program's Common Terminology Criteria for Adverse Events(CTCAE version 5.0)^10^. For patients with multiple diseases, the two largest focus was selected for response assessment. The overall response rate (ORR) was defined as CR+PR. All patients were followed up by calls, outpatient service and hospitalization for 1-43 months, and no patients lost follow-up. The last follow-up date was 2019/6/30. Progression-free survival (PFS) was defined as the time from DEB-TACE treatment to disease progression or patients' death; overall survival (OS) was defined as the time from DEB-TACE treatment to patients' death or the last follow-up date.

### Statistical analysis

Statistical analysis was conducted with the use of SPSS 22.0 statistical software (SPSS Inc., Chicago, USA). Count data were expressed as count (percentage). Survival characteristics were shown using the Kaplan-Meier curve, and the univariate and multivariate Cox's proportional hazards regression analyses were used to determine prognostic factors of OS. *P*-value <0.05 was considered significant.

## Results

### Patients' baseline characteristics

Among the 88 unresectable ICC patients included, 65 were men (73.9%) and 60 patients aged>60 years (68.2%). Fifty-eight percent of patients were ECOG PS 1 and 94.3% Child A in terms of clinical performance status and liver function. There were 25 patients had a postoperative recurrence of the disease. The numbers of patients with previous treatment, subsequent treatment and combined treatment were 28 (31.8%), 29 (33.0%) and 42 (47.7%) respectively. The baseline characteristics of patients are presented in **Table 1**.

### Treatment response

A total of one hundred and twenty-six DEB-TACE procedures were carried out and 1.43 DEB-TACE sessions were carried out per patient (range 1 - 5). Most of them were treated only one time (n=64), only fourteen patients for two and seven for 3 sessions. There were 31 cases combined with TAI of oxaliplatin(n=26) or raltitrexed(n=5). In the posttreatment evaluation and according to mRECIST criteria, 58 patients (65.9 %) met PR criteria, 19 (21.6 %) were SD, and 11 were PD (12.5%) and no CR was observed (**Table 2**). The overall response rate (ORR) to DEB-TACE with CSM in unresectable ICC patients was 65.9%.

### Patients' survival profiles

**Figure 1:**
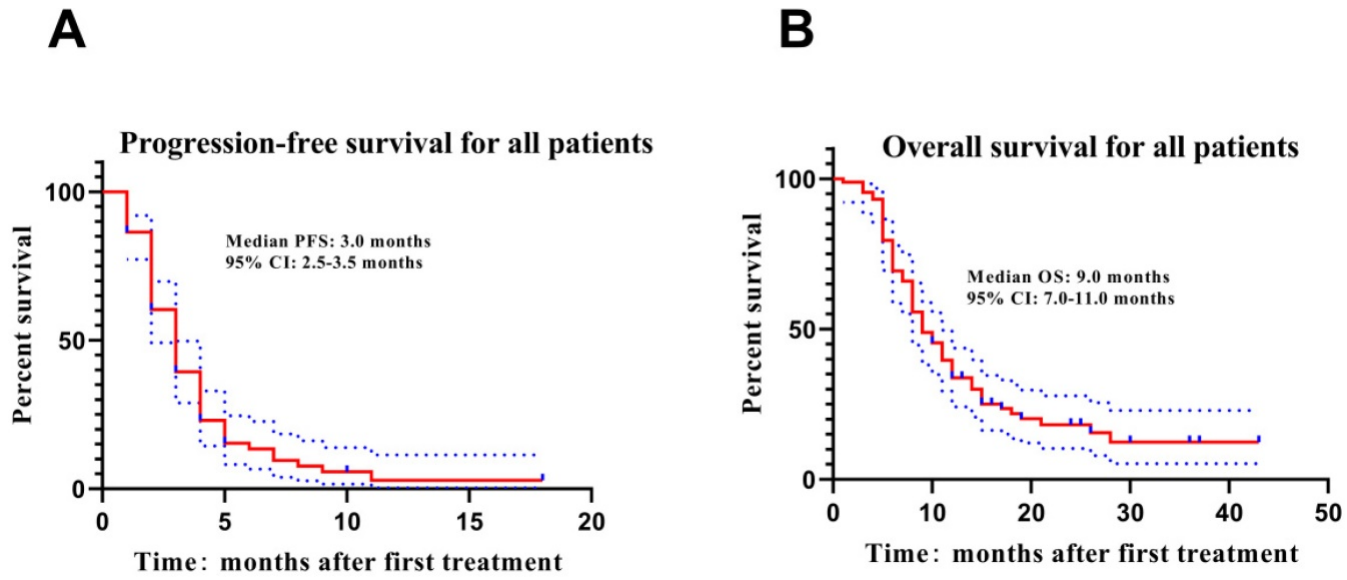
** PFS and OS of ICC patients underwent DEB-TACE with CSM.** The median PFS(A) and OS(B) were 3.0 months and 9.0 months respectively in ICC patients underwent DEB-TACE with CSM treatment. Kaplan-Meier curve was used to evaluate PFS and OS in ICC patients. PFS, progression-free survival; OS, overall survival; ICC intrahepatic cholangiocarcinoma.

Kaplan-Meier curve was used to estimate PFS and OS of unresectable ICC patients underwent DEB-TACE with CSM, which exhibited that the median PFS was 3.0 months and the median OS was 9.0 months (**Figure 1**).

### Factors affecting OS in unresectable ICC patients

**Figure 2:**
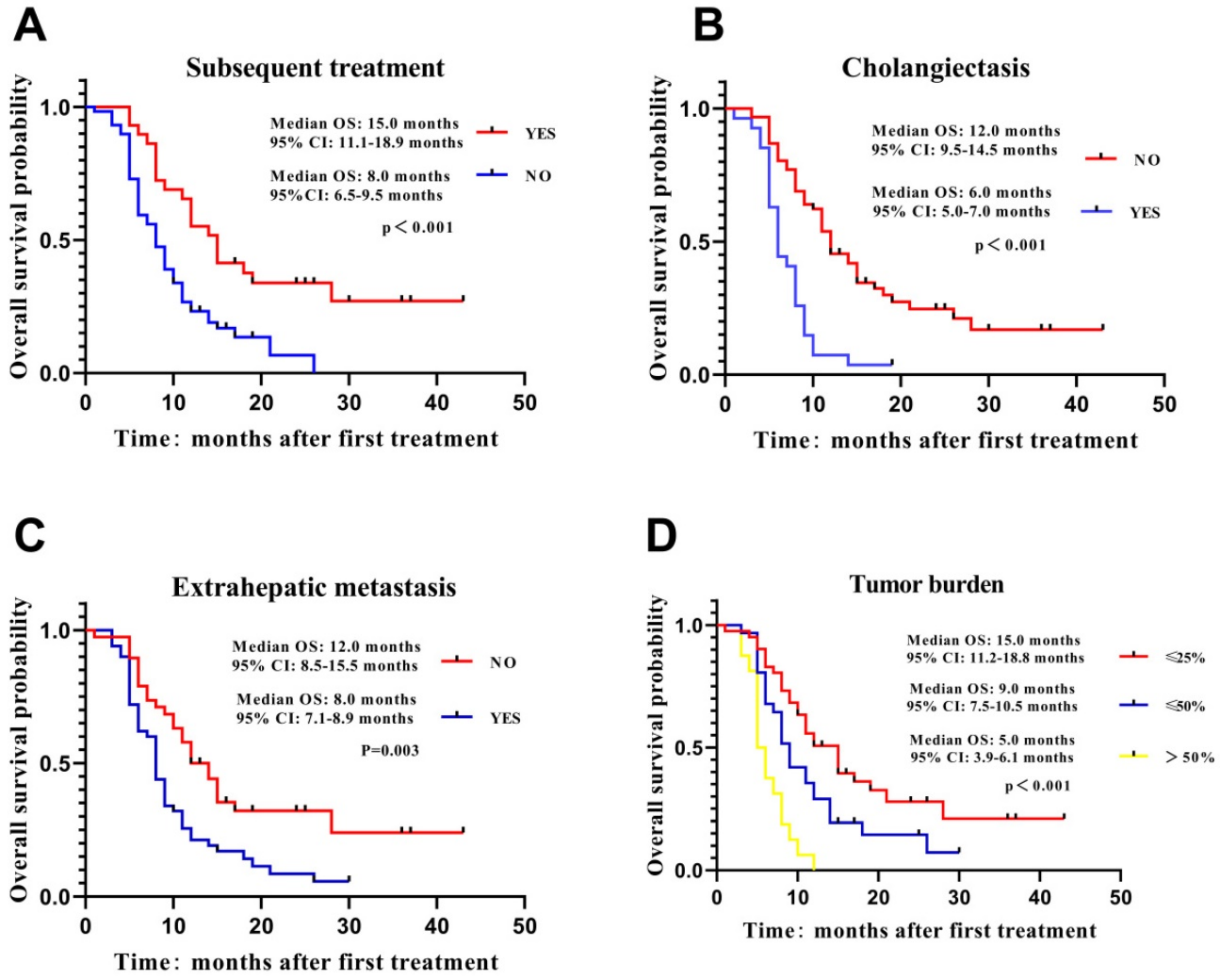
** Comparison of OS in subgroup analysis.** Independent predictive factors for OS were selected in subgroup analysis to further validate their influence on patients' survival. Patients with subsequent treatment (**A**), no cholangiectasis (**B**), extrahepatic metastasis (**C**) and tumor burden (**D**) presented longer OS. Survival characteristics were shown using the Kaplan-Meier curve. OS, overall survival.

Information from 88 unresectable ICC patients treated with DEB-TACE with CSM was included in Cox's proportional hazard regression model analysis (**Table 3**). Univariate regression analysis exhibited that previous treatment (HR=0.491, p=0.010), subsequent treatment (HR=0.408, p=0.001) and combined treatment (HR = 0.445, *P* = 0.001) were correlated with better OS, whereas tumor size (HR = 2.943, *P*<0.001), vascular invasion (HR = 2.467, *P*<0.001), cholangiectasis (HR = 3.215, *P* < 0.001), extrahepatic metastasis (HR = 2.011, *P* = 0.005),CA-125 (HR = 1.658, *P* = 0.042), CA-199 (HR = 2.145, *P* = 0.002) and tumor burden (*P* < 0.001) were associated with worse OS in unresectable ICC patients. Multivariate regression analysis presented that subsequent treatment (HR = 0.519, *P* =0.020) independently predicted longer OS, while cholangiectasis (HR = 2.718, *P* < 0.001), extrahepatic metastasis (HR = 1.776, *P* = 0.033) and tumor burden (*P* = 0.011) independently predicted worse OS in unresectable ICC patients. In addition, factors that independently predicted longer OS were selected for further subgroup analysis, and the result elucidated that OS was better in patients with subsequent treatment (**Figure 2A**), without cholangiectasis (**Figure 2B**) and extrahepatic metastasis (**Figure 2C**) as well as lower tumor burden (**Figure 2D**).

### Adverse events after treatment

After 126 DEB-TACE sessions, the major AEs included vomiting/nausea (77.0%), right upper quadrant pain (61.1%), transient elevation of liver transaminase (54.0%) and low-grade fever (46.0%). One patient occurred myasthenia of the lower limbs for cerebral infarction and fully recovered after neurotrophic treatment for one week. Other complications were resolved 3-5 days after symptomatic supports including analgesia and to stop vomiting. The minor AEs were mainly associated with chemotherapy drugs or post-embolization syndrome. They were mild or moderate, mainly in grades 1-2 (Table 4).

## Discussion

ICC is a highly lethal hepatobiliary neoplasm whose incidence has been increasing steadily and substantially over the last few decades globally. Surgical resection is the potentially curative treatment option for patients with resectable ICC, and arterially directed therapies are generally accepted treatments for unresectable ICC according to the NCCN guidelines.TACE is a promising, minimally invasive intra-arterial therapy for unresectable liver tumors 11. It allows the delivery of high doses of chemotherapeutic drugs directly to the tumor with very little systemic drug exposure and has become a valid alternative to systemic chemotherapy for unresectable ICC 12. Compared with conventional lipiodol-TACE, DEB-TACE is superior to lipiodol in drug loading and releasing as well as embolization effects, is increasingly used in the treatment of ICC 11,13.

Several studies have been published reporting the efficacy of DEB-TACE in treating ICC patients. Research by Aliberti C *et al* 14 revealed that for ICC patients underwent DEB-TACE with DC-Beads or Lifepearls drug-eluting microspheres loaded with doxorubicin, the PR, SD, PD and DCR were 15%, 80%, 5% and 95% at 3 months after treatment according to RECIST 1.1 criteria. Besides, Kuhlmann JB *et al.*^13^ adopted irinotecan-eluting beads treating 26 unresectable ICC patients and revealed that PR, SD and PD rates were 4%, 42% and 50% at 2 months after treatment according to RECIST criteria, while the mean PFS and OS were 3.9 months and 11.7months respectively. Venturini M* et al*^15^ treated 10 patients affected by multiple liver metastases from cholangiocarcinoma with DEB preloaded with irinotecan (DEBIRI) or doxorubicin (DEBDOX) as second-line treatment, resulting in a significantly longer PFS(12.67 weeks for DEBIRI and 15.78 weeks for DEBDOX) and OS (45 weeks in DEBIRI and 48.9 weeks in DEBDOX) after 32 TACE procedures. However, these previous studies were limited in several classical types of DEBs with only a small number of patients and for this reason, clinicians are still skeptical on its efficacy for ICC therapy 13-14.

CSM, which is the first DEB product independently researched and developed in China with good biocompatibility, suspension property, and flexibility, is a type of ion-exchange bead with some negatively charged functional groups. These negatively charged functional groups are responsible for the loading of many positively charged drugs, such as irinotecan and doxorubicin 16-17. In vitro experiments have shown that approximately 90% of doxorubicin can be sequestered into a vial of 100-300 μm CSM within 30 min under given conditions 16. Recently, several clinical studies have reported the safety and effectiveness of DEB-TACE with CSM for the treatment of liver cancer, with the results proved that CSM was efficient and well-tolerated not only in treating patients with HCC but also in secondary liver cancer^11^,^17^.

In this study, 88 unresectable ICC patients received 126 DEB-TACE procedures with CSM loaded with epirubicin. Regarding tumor response, PR was achieved in 58 patients (65.9%), SD in 19 (21.6%) and PD in 11 (12.5%) at 1 month after treatment according to mRECIST criteria. Therefore, the ORR was 65.9%, which were similar or superior rates in previous studies. While the median PFS and OS were 3.0 months and 9.0 months respectively in our cohort, which was numerically lower than that of previous studies. The possible reason was that patients in our study were mainly multiple ICC patients (70.5%) and most of them only received one cycle (72.7%) of DEB-TACE treatment for financial reasons, which might reduce treatment efficacy and influence prognosis of ICC patients. Although these results were not in accordance with previous studies, this is the first study with a relatively larger sample size suggesting its feasibility and effectivity for DEB-TACE with CSM in treating unresectable ICC.

Many studies looked into the prognostic factors that predicted the survival outcomes in ICC. Various studies including the present study have shown extensive intrahepatic tumor burden and extrahepatic metastases to predict poor survival outcomes 18-21. Most extrahepatic metastasis occurs in patients with advanced intrahepatic tumor stage 12. Therefore, the absence of extrahepatic metastasis was associated with a significantly increased median OS 22-23. Zhang XF et al^24^ had explored the prognostic implication of the number and station of lymph node metastasis (LNM) for patients with ICC by using the SEER registry and found that patients with 1 or 2 LNM had comparable worse OS versus patients with no nodal disease (median OS, 1 LNM 18.0, 2 LNM 20.0 vs no LNM 45.0 months, both P < 0.001).In this cohort, the median OS for patients with or without extrahepatic metastasis were 8.0 months and 12.0 months respectively (HR = 1.776, *P* = 0.033). Tumor burden is an important factor in defining prognosis among patients with primary and secondary liver cancers 18,25.Extensive intrahepatic tumor burden might reflect elevated disease severity as well as less hepatic functional reserve, thereby led to poor survival after DEB-TACE treatment in ICC patients^26^. In our study, the median OS for patients with different intrahepatic tumor burden were 15.0 months (≤25%), 9.0 months (≤50%) and 5.0 months (>50%) respectively (P = 0.011).

It is well known that ICC is one of the most refractory and vicious tumors, so a multidisciplinary approach to treatment is necessary. As combining DEB-TACE with systemic/loco-regional treatments may trigger synergistic effects and enhance the efficacy of monotherapy^12^, patients with TACE- subsequent treatments exhibited a higher overall survival rate compared to those with monotherapy (mOS 15.0 months vs 8.0 months, HR = 0.519, *P* =0.020) in our cohort. Cholangiectasis from bile duct stricture due to the tumor, as one of the important clinicopathological appearances of ICC^27^, was closely associated with malignant tumor had infiltrated into the bile duct and increased risk of infection after DEB-TACE treatment, which might worsen OS in HCC patients^27^-^29^. In our study, subgroup analysis showed the median OS for patients with or without cholangiectasis were 12.0 months and 6.0 months respectively (HR = 2.718, *P* < 0.001). As far as we know, it's the first report that revealed the relationship between cholangiectasis and prognosis of ICC, and further researches are needed to further confirm.

Besides treatment response and survival, DEB-TACE has also been reported to be at least as tolerable as traditional TACE in previous studies about liver cancers, and the common adverse events are pain, vomiting, nausea and fever 8,30. As for the safety profiles of DEB-TACE in the treatment of unresectable ICC, the most prevalent adverse events are abdominal pain, nausea, vomiting and transaminase rise 14. In line with this evidence, our study observed that the common adverse events of DEB-TACE with CSM in unresectable ICC patients were mild or moderate post-embolization syndrome (composed of right upper quadrant pain and nausea/vomiting) and chemotherapy-associated toxicities, which demonstrated the relatively good safety of this treatment.

Our study has several important limitations. First, the population size of this study is relatively small, although this is the largest study from a single center with CSM in DEB-TACE treatment for unresectable ICC patients. Second, the follow-up time is relatively short. Therefore, the long-term efficacy and safety of DEB-TACE with CSM in unresectable ICC patients are not assessed and the results must be taken as preliminary. Third, this study has not a control group. We are currently enrolling patients into a randomized controlled phase II study, and the results from this single-group preliminary study can be further validated in the randomized controlled phase II study.

## Conclusion

Although the present preliminary study is clearly limited by its retrospective and nonrandomized study design, this is the first report demonstrates that DEB-TACE with CSM is safe and well-tolerated for unresectable ICC patients, with a low complication rate and a relative benefit in terms of survival. Subsequent treatments including systemic/loco-regional treatments is an independent favorable prognostic factor, but cholangiectasis, extensive intrahepatic tumor burden and extrahepatic metastases are the three prognostic factors associated with poor survival. On the whole, our preliminary research shows a promising perspective for future randomized controlled phase II study that will focus on the effectiveness and safety of CSM in unresectable ICC patients.

## Figures and Tables

**Table 1:** Patients' baseline characteristics (N=88)

Characteristics	No.	Percentage
Age (years)		
≤60	28	31.8
>60	60	68.2
Gender		
Male	65	73.9
Female	23	26.1
ECOG performance status		
0	37	42.0
1	51	58.0
Child-Pugh classification		
A	83	94.3
B	5	5.7
Postoperative recurrence		
Yes	18	20.5
No	70	79.5
Previous treatment		
Yes	28	31.8
No	60	68.2
Subsequent treatment		
Yes	29	33.0
No	59	67.0
Combined treatment		
Yes	42	47.7
No	46	52.3
TAI		
Yes	31	35.2
No	57	64.8
Strengthen embolization		
Yes	19	21.6
No	69	78.4
No. of tumor		
Solitary	26	29.5
Multiple	62	70.5
Tumor location		
Unilateral	59	67.0
Bilateral	29	33.0
Liver tumor burden		
≤25%	41	46.6
>25% ≤50%	31	35.2
>50%	16	18.2
Largest diameter (cm)		
≤5	32	36.4
>5	56	63.6
Cholangiectasis		
Yes	27	30.7
No	61	69.3
Serum CA-125 (U/ml)		
≤35	57	64.8
>35	31	35.2
Serum CA-199 (U/ml)		
≤37	42	47.7
>37	46	52.3
Extrahepatic disease		
Yes	50	56.8
No	38	43.2
Vascular invasion		
Yes	34	38.6
No	54	61.4

Data were presented as count and percentage. ECOG, Eastern Cooperative Oncology Group; CA-125, carbohydrate antigen 125; CA-199, carbohydrate antigen 199; TAI, transcatheter arterial infusion.

**Table 2:** Treatment response

Response	Patients (n/%)
CR	0 (0.0)
PR	58 (65.9)
SD	19 (21.6)
PD	11 (12.5)
ORR	57 (65.9)

Data were presented as count (percentage). CR, complete remission; PR, partial remission; SD, stable disease; PD, progression disease; ORR, overall response rate (ORR=CR+PR).

**Table 3:** Cox's proportional hazards regression model analysis of factors affecting OS.

Variable	Univariate analysis			Multivariate analysis		
	HR	95% CI	*P*	HR	95% CI	*P*
Gender (Male/Female)	0.754	0.442-1.289	0.302	-	-	-
TAI (Yes/No)	0.884	0.540-1.448	0.625	-	-	-
Strengthen embolization (Yes/No)	1.442	0.834-2.495	0.190	-	-	-
Previous treatment (Yes/No)	0.491	0.286-0.841	0.010	-	-	-
Subsequent treatment (Yes/No)	0.408	0.236-0.703	0.001	0.519	0.298-0.903	0.020
Combined treatment (Yes/No)	0.445	0.272-0.728	0.001	-	-	-
Tumor location(Unilateral/Bilateral)	1.025	0.623-1.687	0.922	-	-	-
Tumor No. (1/≥2)	1.247	0.749-2.078	0.396	-	-	-
Tumor size (≤5 cm/>5 cm)	2.943	1.682-5.147	0.000	-	-	-
Vascular invasion (Yes/No)	2.467	1.517-4.011	0.000	-	-	-
Extrahepatic metastasis (Yes/No)	2.011	1.232-3.280	0.005	1.776	1.049-3.008	0.033
Cholangiectasis (Yes/No)	3.215	1.913-5.403	0.000	2.718	1.597-4.626	0.000
CA-125 (≤35 U/ml />35 U/ml)	1.658	1.019-2.700	0.042	-	-	-
CA-199 (≤37 U/ml />37 U/ml)	2.145	1.314-3.502	0.002	-	-	-
Tumor burden			0.000			0.011
≤25%	Ref			Ref		
>25%, ≤50%	1.763	1.035-3.005	0.037	1.274	0.724-2.240	0.401
>50%	4.703	2.429-9.107	0.000	2.953	1.482-5.884	0.002

Data were presented as P value, HR (hazards ratio) and 95% CI (confidence interval). Factors affecting OS (overall survival) were determined by univariate and multivariate Cox's proportional hazards regression model analyses. *P*-value <0.05 was considered significant. CA-125, carbohydrate antigen 125; CA-199, carbohydrate antigen 199; TAI, transcatheter arterial infusion.

**Table 4:** Adverse events occurred after DEB-TACE treatment (126 DEB-TACE records).

Parameters	n (%)	Grade
Vomiting/Nausea	97/126 (77.0)	1-2
Abdominal pain	77/126 (61.1)	1-2
Aminotransferase elevation	68/126 (54.0)	1-2
Low-grade fever	58/126 (46.0)	1
Decreased strength of the lower limbs for cerebral infarction	2/126 (1.6)	2

Data were presented as count (percentage). The description was based on 126 DEB-TACE records. DEB-TACE, drug-eluting bead transarterial chemoembolization.

## Abbreviations

DEB-TACE: Drug-eluting beads transcatheter arterial chemoembolization
TACE: Transcatheter arterial chemoembolization
TAI: Transcatheter arterial infusion
CSM: CalliSpheres^®^ microsphere
ICC: Intrahepatic cholangiocarcinoma
mRECIST: modified Response Evaluation Criteria in Solid Tumors
AEs: Adverse events
OS: Overall survival
CR: Complete response
ECOG: Eastern Cooperative Oncology Group
ORR: Overall response rate
PFS: Progression free survival
PR: Partial response
SD: Stable disease
PD: Progressive disease
CT: Computed Tomography
MRI: Magnetic Resonance Imaging
DSA: Digital Subtraction Angiography

## References

[1] Mazzaferro V, Gorgen A, Roayaie S (2020). Liver resection and transplantation for intrahepatic cholangiocarcinoma. J Hepatol.

[2] Bray F, Ferlay J, Soerjomataram I (2018). Global cancer statistics 2018: GLOBOCAN estimates of incidence and mortality worldwide for 36 cancers in 185 countries. CA Cancer J Clin.

[3] Hu LS, Zhang XF, Weiss M (2019). Recurrence Patterns and Timing Courses Following Curative-Intent Resection for Intrahepatic Cholangiocarcinoma. Ann Surg Oncol.

[4] Wang ML, Ke ZY, Yin S (2019). The effect of adjuvant chemotherapy in resectable cholangiocarcinoma: A meta-analysis and systematic review. Hepatobiliary Pancreat Dis Int.

[5] Shao G, Liu R, Ding W (2018). Efficacy and safety of raltitrexed-based transarterial chemoembolization for colorectal cancer liver metastases. Anticancer Drugs.

[6] Chen ZH, Zhang XP, Zhou TF (2019). Adjuvant transarterial chemoembolization improves survival outcomes in hepatocellular carcinoma with microvascular invasion: A systematic review and meta-analysis. Eur J Surg Oncol.

[7] Xiang X, Lau WY, Wu ZY (2019). Transarterial chemoembolization versus best supportive care for patients with hepatocellular carcinoma with portal vein tumor thrombusa multicenter study. Eur J Surg Oncol.

[8] Sun J, Zhou G, Zhang Y (2018). Comprehensive analysis of common safety profiles and their predictive factors in 520 records of liver cancer patients treated by drug-eluting beads transarterial chemoembolization. Medicine (Baltimore).

[9] Kis B, El-Haddad G, Sheth RA (2017). Liver-Directed Therapies for Hepatocellular Carcinoma and Intrahepatic Cholangiocarcinoma. Cancer Control.

[10] Lucatelli P, Ginnani CL, De Rubeis G (2019). Balloon-Occluded Transcatheter Arterial Chemoembolization (b-TACE) for Hepatocellular Carcinoma Performed with Polyethylene-Glycol Epirubicin-Loaded Drug-Eluting Embolics: Safety and Preliminary Results. Cardiovasc Intervent Radiol.

[11] Peng Z, Cao G, Hou Q (2019). The comprehensive analysis of efficacy and safety of CalliSpheres(R) drug-eluting beads transarterial chemoembolization in 367 liver cancer patients: a multiplecenter, cohort study. Oncol Res.

[12] Gusani NJ, Balaa FK, Steel JL (2008). Treatment of unresectable cholangiocarcinoma with gemcitabine-based transcatheter arterial chemoembolization (TACE): a single-institution experience. J Gastrointest Surg.

[13] Kuhlmann JB, Euringer W, Spangenberg HC (2012). Treatment of unresectable cholangiocarcinoma: conventional transarterial chemoembolization compared with drug eluting bead-transarterial chemoembolization and systemic chemotherapy. Eur J Gastroenterol Hepatol.

[14] Aliberti C, Carandina R, Sarti D (2017). Chemoembolization with Drug-eluting Microspheres Loaded with Doxorubicin for the Treatment of Cholangiocarcinoma. Anticancer Res.

[15] Venturini M, Sallemi C, Agostini G (2016). Chemoembolization with drug eluting beads preloaded with irinotecan (DEBIRI) vs doxorubicin (DEBDOX) as a second line treatment for liver metastases from cholangiocarcinoma: a preliminary study. Br J Radiol.

[16] Zhang S, Huang C, Li Z (2017). Comparison of pharmacokinetics and drug release in tissues after transarterial chemoembolization with doxorubicin using diverse lipiodol emulsions and CalliSpheres Beads in rabbit livers. Drug Deliv.

[17] Zhang X, Zhou J, Zhu DD (2019). CalliSpheres(R) drug-eluting beads (DEB) transarterial chemoembolization (TACE) is equally efficient and safe in liver cancer patients with different times of previous conventional TACE treatments: a result from CTILC study. Clin Transl Oncol.

[18] Bagante F, Spolverato G, Merath K (2019). Intrahepatic cholangiocarcinoma tumor burden: A classification and regression tree model to define prognostic groups after resection. Surgery.

[19] Kohler M, Harders F, Lohofer F (2019). Prognostic Factors for Overall Survival in Advanced Intrahepatic Cholangiocarcinoma Treated with Yttrium-90 Radioembolization. J Clin Med.

[20] Seo JW, Kwan BS, Cheon YK (2020). Prognostic impact of hepatitis B or C on intrahepatic cholangiocarcinoma. Korean J Intern Med.

[21] Zhu YJ, Xu Q, Shao MY (2019). Decreased expression of HDAC8 indicates poor prognosis in patients with intrahepatic cholangiocarcinoma. Hepatobiliary Pancreat Dis Int.

[22] Sha M, Jeong S, Wang X (2019). Tumor-associated lymphangiogenesis predicts unfavorable prognosis of intrahepatic cholangiocarcinoma. BMC Cancer.

[23] Gangi A, Shah J, Hatfield N (2018). Intrahepatic Cholangiocarcinoma Treated with Transarterial Yttrium-90 Glass Microsphere Radioembolization: Results of a Single Institution Retrospective Study. J Vasc Interv Radiol.

[24] Zhang XF, Xue F, Dong DH (2020). Number and Station of Lymph Node Metastasis After Curative-intent Resection of Intrahepatic Cholangiocarcinoma Impact Prognosis. Ann Surg.

[25] Ruzzenente A, Bagante F, Ratti F (2019). Response to preoperative chemotherapy: impact of change in total burden score and mutational tumor status on prognosis of patients undergoing resection for colorectal liver metastases. HPB (Oxford).

[26] Palmer DH, Hawkins NS, Vilgrain V (2020). Tumor burden and liver function in HCC patient selection for selective internal radiation therapy: SARAH post-hoc study. Future Oncol.

[27] Xu CC, Tang YF, Ruan XZ (2017). The value of Gd-BOPTA- enhanced MRIs and DWI in the diagnosis of intrahepatic mass-forming cholangiocarcinoma. Neoplasma.

[28] Fujita N, Asayama Y, Nishie A (2017). Mass-forming intrahepatic cholangiocarcinoma: Enhancement patterns in the arterial phase of dynamic hepatic CT - Correlation with clinicopathological findings. Eur Radiol.

[29] Zheng BH, Yang LX, Sun QM (2017). A New Preoperative Prognostic System Combining CRP and CA199 For Patients with Intrahepatic Cholangiocarcinoma. Clin Transl Gastroenterol.

[30] Zhou GH, Han J, Sun JH (2018). Efficacy and safety profile of drug-eluting beads transarterial chemoembolization by CalliSpheres(R) beads in Chinese hepatocellular carcinoma patients. BMC Cancer.

